# Utility of Ischemia Testing Prior to Ablation for Sustained Monomorphic Ventricular Tachycardia

**DOI:** 10.19102/icrm.2022.130301

**Published:** 2022-03-15

**Authors:** Nishaki K. Mehta, Christopher Schumann, Giovanni Davogustto, Andrew Cluckey, Evan Harmon, Joshua France, James M. Mangrum, Pamela Mason, Sula Mazimba, Rohit Malhotra, Kenneth Bilchick, Andrew Darby, Michael Salerno, Christopher M. Kramer, William Stevenson

**Affiliations:** ^1^Division of Cardiovascular Medicine, Beaumont Hospital, Royal Oak, MI, USA; ^2^Department of Medicine, University of Virginia Health System, Charlottesville, VA, USA; ^3^Cardiovascular Division, Department of Medicine, Vanderbilt University Medical Center, Nashville, TN, USA; ^4^Department of Radiology and Medical Imaging, University of Virginia Health System, Charlottesville, VA, USA; ^5^Department of Biomedical Engineering, University of Virginia Health System, Charlottesville, VA, USA

**Keywords:** Catheter ablation, coronary artery disease, ischemia testing, revascularization, ventricular tachycardia

## Abstract

The aim of this study was to determine the relationship between ischemia testing prior to ablation for sustained monomorphic ventricular tachycardia (VT) (SMVT) and post-ablation mortality and VT recurrence. As SMVT is generally caused by myocardial scar and not active ischemia, the utility of ischemia testing prior to SMVT ablation is unclear. Patients who underwent ablation for SMVT at 2 tertiary care centers between January 2016 and July 2018 were included in a retrospective study. A Kaplan–Meier survival analysis was performed, stratifying patients by pre-ablation ischemia testing for the endpoints of mortality and VT recurrence. A Cox multivariable regression analysis was performed to identify predictors of post-ablation VT recurrence. A total of 163 patients were included, with 46 (28%) patients undergoing ischemia testing prior to ablation. Only 5 of the 46 patients (11%) received revascularization pre-ablation. After a median follow-up period of 625 days (interquartile range, 292–982 days) following ablation, 97 of 163 patients (60%) had VT recurrence, and 32 patients (20%) had died. There was no difference in mortality or VT recurrence between patients who did or did not experience ischemia testing or revascularization. In the multivariable regression analysis, predictors of VT recurrence were the number of anti-arrhythmics failed, non-ischemic cardiomyopathy, sex, and cardiac magnetic resonance imaging pre-ablation. Neither ischemia testing nor revascularization was a significant predictor of VT recurrence in univariable or multivariable regression analysis. In conclusion, ischemia testing is frequently ordered prior to SMVT ablation but infrequently leads to revascularization and is not associated with post-ablation outcomes. The findings support adopting an individualized approach rather than performing routine ischemia testing.

## Introduction

Sustained monomorphic ventricular tachycardia (VT) (SMVT) is a potentially life-threatening arrhythmia that is generally caused due to re-entry in a region of myocardial scar in patients with structural heart disease.^1^ Catheter ablation is used to reduce the recurrence of SMVT by disrupting conduction channels within a myocardial scar^2^ and is superior to anti-arrhythmic drug and implantable cardioverter-defibrillator (ICD) therapies in preventing VT recurrence.^3–5^

While catheter ablation is an effective treatment, the optimal pre-ablation work-up of patients presenting with SMVT remains unclear. Coronary artery disease (CAD) is the most common cause of both myocardial scar and sudden death from arrhythmias^6^ such that the presence of an arrhythmia often elicits an assessment for myocardial ischemia. Assessment of the severity of CAD by angiography, including computed tomography angiography (CTA) or invasive coronary angiography, or ischemia evaluation by non-invasive stress testing is often recommended prior to ablation to rule out ischemia as the cause of VT.^7^ However, as SMVT is usually caused by re-entry resulting from scarring and not active ischemia, the utility of ischemia testing is unclear. The goals of this study were to evaluate the prevalence of ischemia testing prior to ablation in patients presenting with SMVT and to determine whether ischemia testing or revascularization affects post-ablation outcomes of mortality or VT recurrence.

## Methods

### Study population

Our study was a retrospective investigation focused on patients who underwent catheter ablation for SMVT between January 2016 and July 2018 at 2 tertiary care centers (University of Virginia Medical Center and Vanderbilt University Medical Center). All patients who underwent elective ablation for SMVT were included, including those with prior ablations. Baseline clinical and demographic data were recorded. All patients were assessed for whether ischemia testing was performed prior to ablation, specifically within 30 days of the procedure. Additional analyses were performed for patients who underwent ischemia testing within 1 year of their ablation procedure. Ischemia testing included invasive coronary angiography, CTA, or non-invasive stress testing. For patients who underwent ischemia testing, the results of the test and whether they underwent revascularization were recorded. Patients were also assessed for whether they had cardiac magnetic resonance (CMR) imaging at any point prior to their ablation. To improve the quality of our data collection, we not only reviewed the electronic medical record but also systematically screened online obituaries to accurately report outcomes of mortality and VT recurrence. From the review of electronic medical records and online obituaries,^8^ outcomes of mortality and VT recurrence were assessed. Recurrent sustained VT was determined by in-hospital records and a review of all subsequent ICD interrogations. Recurrence during the follow-up period was defined as any episode of monomorphic VT lasting >30 seconds and/or requiring ICD therapy. Patients were censored at the time of recurrent VT, death, or last clinical contact. This research was compliant with ethical standards, and the study was approved by the institutional review boards at both institutions.

### Statistical analysis

Statistical analysis was performed using SAS version 9.4 (SAS Institute, Cary, NC, USA). Patients were stratified by pre-ablation ischemia testing. Continuous variables are reported as mean ± standard deviation values, while categorical variables are reported as percentages. Statistical significance was assessed using Student’s *t*-test for continuous variables and chi-squared/Fisher’s exact tests for categorical variables, with *P* < .05 being considered significant. A Kaplan–Meier survival analysis was performed, stratifying patients by pre-ablation ischemia testing and revascularization for the endpoints of mortality and VT recurrence. To identify predictors of VT recurrence following ablation, a Cox regression analysis was performed to determine hazard ratios (HRs), modeling the time to first occurrence of VT after ablation. A stepwise multivariable analysis was performed in which variables that produced *P* < .05 were included in the final model.

## Results

### Baseline characteristics/prevalence of ischemia testing

**Figure 1:**
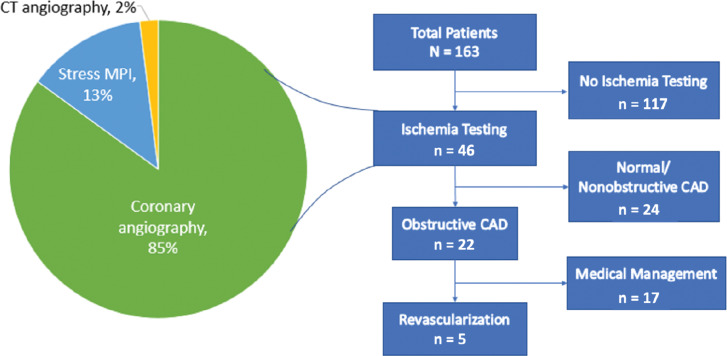
Study population flowchart. Ischemia testing was ordered in 46 of 163 patients within 30 days of ventricular tachycardia ablation. Of the 46 patients, 22 were found to have obstructive coronary artery disease, but only 5 received revascularization prior to ablation. *Abbreviations:* CAD, coronary artery disease; CT, computed tomography; MPI, myocardial perfusion imaging.

A total of 163 patients (62.4 ± 13.8 years, 12.3% female) were included, with 46 (28%) patients having undergone ischemia testing prior to ablation. Of the total patients, 107 (65%) had an ischemic cardiomyopathy, 52 (32%) had a non-ischemic cardiomyopathy, and 4 (2%) were unspecified. The number of patients who had a prior VT ablation was 41 (25%). Compared to those who did not undergo ischemia testing, patients who had ischemia testing had higher rates of hypertension, were more likely to have presented with VT storm, were prescribed spironolactone less often, and were less likely to have failed amiodarone **(Table 1)**. Of the 46 patients who underwent ischemia testing, coronary angiography was performed in 39 patients (85%), with 6 of these patients undergoing angiography during epicardial ablation for safety purposes. Of the remaining patients, six underwent stress myocardial perfusion imaging (13%) and one underwent CTA (2%). Of the 46 patients who underwent ischemia testing, 22 (48%) were found to have obstructive CAD. Only 5 of the 46 patients (11%) received revascularization prior to VT ablation **(Figure 1)**. These 5 patients did not undergo stress testing prior to coronary angiography that led to revascularization. All 5 patients had a history of CAD, and 2 had undergone coronary artery bypass grafting. Three of the 5 patients who were revascularized had presented with VT storm. Each ablation in the study was performed with radiofrequency energy (161 ablations), with 2 exceptions being surgical cryoablation and alcohol ablation. Twenty of the 163 ablations were epicardial in nature. Of the 163 ablations included in the study, 9 procedures utilized hemodynamic support with the following techniques: left ventricular assist device (n = 7), Impella (Abiomed, Danvers, MA, USA) (n = 1), and cardiopulmonary bypass (n = 1).

### Procedure outcomes

A total of 163 ablation procedures were performed, with a median procedure time of 219.5 minutes (interquartile range [IQR], 164–278 min). The median number of morphologies of VT that were ablated per procedure was 2 (IQR, 1–2). VT remained inducible at the end of the procedure in 35 of 163 cases (21.4%). Complications were reported in 23 of 163 cases (14.1%), including pericardial effusion/tamponade (5 cases, 2 with epicardial access for ablation); femoral access bleeding, fistulae, or pseudoaneurysm (5 cases); left bundle branch block (2 cases); complete heart block (2 cases); respiratory failure (2 cases); pericarditis (2 cases); stroke (1 case); and lower extremity embolus (1 case). There was 1 death due to tamponade.

### Ventricular tachycardia recurrence and mortality outcomes

**Figure 2:**
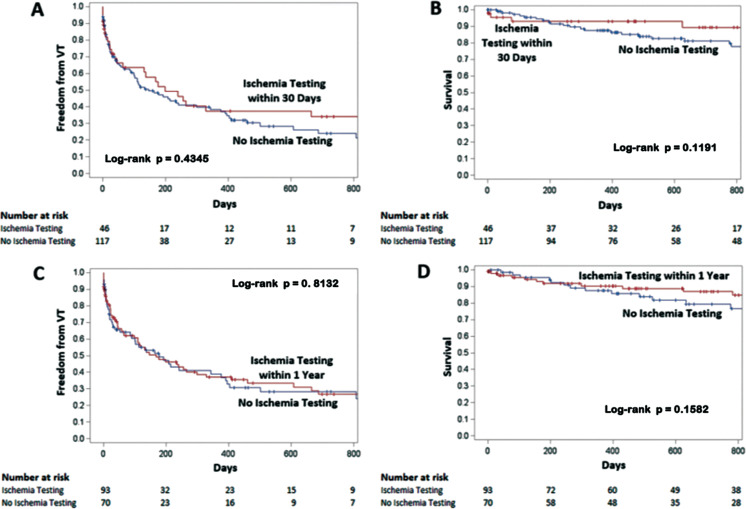
Impact of ischemia testing prior to ventricular tachycardia (VT) ablation on post-ablation outcomes. Kaplan–Meier curves compare pre-ablation ischemia evaluation (defined as within 30 days of the procedure) versus no ischemia testing for **(A)** VT-free survival and **(B)** mortality. There was no difference between the groups for VT-free survival (*P* = .4345) or mortality (*P* = .1191). Extending the ischemia evaluation to within one year of VT ablation, Kaplan–Meier curves compare pre-ablation ischemia testing versus no testing for **(C)** VT-free survival and **(D)** mortality. There was no difference between the groups for VT-free survival (*P* = .8132) or mortality (*P* = .1582).

Patients were followed up with for a median of 625 days (IQR, 292–982 days). VT recurrence occurred in 97 of 163 patients (60%) at a median of 44 days (IQR, 8–178 days), and 32 patients (20%) died post-ablation at a median of 354 days (IQR, 136–807 days). Kaplan–Meier survival analysis showed no difference in mortality (*P* = .1191) or VT recurrence (*P* = .4345) between patients who did and did not undergo ischemia testing 30 days prior to ablation **(Figure 2)**. Extending the analysis of ischemia testing to 1 year prior to the ablation, the total number of patients who underwent testing within 1 year was 92 (56%). There was no difference between the group undergoing testing within 1 year and those who did not in terms of VT-free survival or mortality. There remained no significant difference in mortality or VT recurrence in patients undergoing ischemia testing after excluding the 6 patients who underwent coronary angiography during an epicardial ablation procedure. In addition, there was no difference in mortality (*P* = .8469) or VT recurrence (*P* = .3942) between patients who underwent revascularization prior to the ablation and those who did not.

### Predictors of ventricular tachycardia recurrence

Multivariable Cox proportional hazards analysis identified four significant predictors of VT recurrence post-ablation **(Table 2)**: (1) type of cardiomyopathy (for ischemic, HR, 2.03; 95% confidence interval [CI], 125–3.30); (2) CMR pre-ablation (HR, 0.53; 95% CI, 0.29–0.98); (3) number of failed anti-arrhythmics (HR, 1.46 per drug; 95% CI, 1.23–1.73); and (4) female sex (HR, 0.32; 95% CI, 0.14–0.74). Ischemia testing pre-ablation was not a significant predictor of VT recurrence in univariable analysis (HR, 0.83; 95% CI, 0.53–1.32; (*P* = .4369) or when it was added to the multivariable model **(Table 2)**.

### Sub-analysis of ischemic cardiomyopathy patients

In the ischemic cohort of 107 patients with ischemic heart disease, 35 patients underwent ischemic testing and 5 of the 35 patients underwent revascularization after ischemic testing. Of the initial 107 patients, 60 patients had VT recurrence and 24 patients died. There was no difference in mortality or VT recurrence between patients who had ischemic testing and those who underwent revascularization. Multivariate analysis predictors of VT recurrence were the number of anti-arrhythmics failed and improved outcomes for female patients **(Table 3)**.

## Discussion

### Utility of ischemia testing

Ischemia testing prior to ablation was frequently performed in our study (28%), but revascularization prior to ablation was rarely needed (11% of the subgroup of patients undergoing ischemia testing). Furthermore, neither ischemia testing nor revascularization was associated with different post-ablation outcomes of VT recurrence or mortality. This relates to the mechanism of SMVT in patients with structural heart disease, which can be caused by scar-related reentry and is not driven by active ischemia.^9^ Our findings support the idea that clinicians caring for patients with SMVT should continue to individualize the decision of ischemic evaluation prior to VT ablation.

Prior studies have examined the role of ischemia testing and revascularization in patients with SMVT. Cuk et al. studied 54 patients with CAD who presented with an initial episode of SMVT; of these, 20 patients (37%) underwent revascularization, and the authors found that revascularization had no effect on VT recurrence or mortality at 1 year.^10^ Elsokkari et al. evaluated patients with ischemic cardiomyopathy and VT and found that prior revascularization was not associated with improvement in mortality or VT recurrence.^11^ However, there have been case reports of incessant SMVT resolving with revascularization.^12^ Furthermore, Brugada et al. evaluated patients with a history of myocardial infarction, ventricular arrhythmias not related to acute ischemia, and coronary lesions needing revascularization. These patients underwent an electrophysiology study both before and after revascularization, and the authors found that a small subset of patients (5/53 patients) who had inducible VT prior to revascularization were no longer inducible after revascularization.^13^ These findings suggest that, while the majority of patients would not benefit from revascularization to treat SMVT, there may be a subset who would benefit. Further research is needed to determine how to identify those patients who would benefit from revascularization.

While ischemia is not the cause of SMVT, assessment of the potential for ischemia is undoubtedly warranted prior to catheter ablation procedures in some patients. Patients are at risk for demand ischemia from induction of VT that is commonly performed during the procedure. Hemodynamic instability can occur, increasing patient mortality.^14^ However, our data suggest that significant CAD requiring revascularization pre-ablation is infrequent in this population, as only 5 of the 46 patients (11%) undergoing ischemia testing underwent revascularization. Our findings support an approach to ischemic testing pre-ablation similar to that for preoperative evaluations for noncardiac surgery where, in the absence of symptoms suggestive of ischemia in a patient with adequate functional capacity, ischemia testing is not mandatory. However, a randomized trial would be required to assess the procedural impact of ischemia evaluation.

### Predictors of ventricular tachycardia recurrence

The significant predictors of VT recurrence following ablation in our study were (1) number of failed anti-arrhythmic drugs, (2) type of cardiomyopathy, (3) sex, and (4) pre-ablation CMR. Failure of multiple anti-arrhythmic drugs has been associated with increased rates of VT recurrence post-ablation, as it often indicates more advanced structural heart disease with a refractory arrhythmia substrate.^15^ Type of cardiomyopathy is also a known predictor of ablation success, as patients with non-ischemic cardiomyopathy have been found to have worse outcomes, likely owing to mid-myocardial or epicardial substrates, which are challenging to target.^16^

Female sex was associated with improved outcomes on our multivariable analysis. In a large retrospective study, Frankel et al. noted increased VT recurrence following ablation in women, which they postulated could be due to differences in referral pattern, arrhythmia substrate, or treatment following ablation.^17^ In our study, while the small sample size precludes definitive conclusions, better outcomes in women may be due to earlier referral and intervention, as women were less likely than men to have an ICD (70% vs. 94%, *P* = .0003), less likely to present with VT storm (5% vs. 26%, *P* = .0464), and had a higher ejection fraction (41% ± 15% vs. 29% ± 13%, *P* = .0003).

CMR pre-ablation was associated with an improvement in ablation outcomes in our study. This may be due to the use of late gadolinium enhancement imaging for the detection of myocardial scar,^18^ which can facilitate preprocedural planning of VT ablation. Given that VT ablation procedures are generally performed in patients with compromised cardiac function, and the procedure involves induction of VT, efficient targeting can improve procedure efficiency and outcomes with a reduction in periprocedural complications. CMR guidance for VT ablation has been shown to result in improved outcomes. Andreu et al. studied 159 patients undergoing CMR-guided versus standard ablation and found that the CMR-guided group had a significantly lower rate of VT recurrence (HR, 0.48; 95% CI, 0.24–0.96; *P* = .037),^19^ similar to our findings (HR, 0.53; 95% CI, 0.29–0.98).

### Study limitations

This study has several limitations. First, it is retrospective and therefore only hypothesis-generating. Only patients who underwent VT ablation were identified for inclusion, and likely there were some patients with SMVT who underwent ischemia evaluation and did not receive ablation. However, these patients are difficult to identify retrospectively. Additionally, the cause of death cannot be conclusively ascertained. Further, the recurrence rate of VT observed in this study is higher compared to recent reports.^2^ It is possible that inclusion of patients with non-ischemic cardiomyopathy and repeat ablations in this study may account at least in part for the higher recurrence observed.

In addition, VT recurrence depends on several factors, including but not limited to the timing of ablation, uniform use of procedural technique and endpoints, programming strategies for VT detection, and the use of novel pharmacologic agents like sacubitril not routinely available during this study.^20–22^ However, we believe that the strength of the study is the real-life variability, which is reflective of everyday practice.

To fully characterize the impact of ischemia testing on patients with SMVT, a prospective trial randomizing all patients presenting with SMVT to ischemia testing versus no testing would be needed.

## Conclusion

In this 2-center retrospective analysis of 163 patients undergoing ablation for SMVT, ischemia testing was frequently ordered prior to ablation but infrequently led to revascularization. Neither ischemia testing nor revascularization was associated with outcomes of mortality or VT recurrence. These results suggest that ischemia testing should be individualized pre-ablation, and further research is needed to improve preprocedural evaluation. In particular, prospective randomized trials are desirable to further assess the utility of ischemia testing prior to SMVT ablation.

## Figures and Tables

**Table 1:** Baseline Clinical Characteristics

Clinical Characteristics	Total Cohort n (%)	Ischemia Testing n (%)	No Ischemia Testing n (%)	p-value
Total patients	163	46 (28.2)	117 (71.8)	—
Age, years	62.4 ± 13.8	63.7 ± 13.3	61.8 ± 14.0	0.43
Female	20 (12.3)	7 (15.2)	13 (11.1)	0.47
Caucasian (n = 160)	147 (91.9)	42 (95.5)	105 (90.5)	0.52
BMI	30.0 ± 6.4	29.7 ± 6.4	30.0 ± 6.4	0.76
Diabetes	48 (29.5)	12 (26.1)	36 (30.8)	0.55
Hyperlipidemia	111 (68.1)	32 (69.6)	79 (67.5)	0.80
Hypertension	111 (68.1)	37 (80.4)	74 (63.3)	0.03*
COPD	18 (11.0)	6 (13.0)	12 (10.3)	0.61
CVA	16 (9.8)	5 (10.9)	11 (9.4)	0.77
Smoking	71 (43.6)	25 (54.4)	46 (39.3)	0.08
PAD	15 (9.2)	4 (8.7)	11 (9.4)	1.00
Creatinine	1.2 ± 0.4	1.2 ± 0.5	1.2 ± 0.4	0.59
Dialysis	1 (0.6)	0 (0)	1 (0.9)	1.00
CAD	111 (68.1)	35 (76.1)	76 (65.0)	0.17
CABG	47 (28.8)	13 (28.3)	34 (29.1)	0.92
PCI (n = 152)	76 (50.0)	23 (53.5)	53 (48.6)	0.59
Atrial fibrillation/flutter	78 (47.9)	18 (39.1)	60 (51.3)	0.16
Ischemic cardiomyopathy	107 (65.6)	35 (76.0)	72 (61.5)	0.09
Nonischemic cardiomyopathy	52 (31.9)	10 (21.7)	42 (35.9)	0.09
No cardiomyopathy	4 (2.4)	0 (0)	4 (3.4)	1.00
NYHA class (n = 103)	2.2 ± 0.9	2.1 ± 0.8	2.2 ± 0.9	0.46
LVEF (n = 162)	30.1 ± 14.0	31.3 ± 13.2	29.6 ± 14.3	0.49
Prior VT ablation	41 (25.2)	9 (19.6)	32 (27.4)	0.30
VT storm	38 (23.3)	20 (43.5)	18 (15.4)	0.0001*
CMR pre-ablation	29 (17.8)	8 (17.4)	21 (18.0)	0.93
Aspirin	117 (71.8)	36 (78.3)	81 (69.2)	0.25
P2Y12	44 (27.0)	13 (28.3)	31 (26.5)	0.82
β-Blocker	148 (90.8)	44 (95.7)	104 (88.9)	0.18
ACE/ARB	103 (63.2)	29 (63.0)	74 (63.3)	0.98
Aldactone	67 (41.1)	11 (23.9)	56 (47.9)	0.005*
Statin	107 (65.6)	31 (67.4)	76 (65.0)	0.77
Amiodarone	99 (60.7)	29 (63.0)	70 (59.8)	0.71
Failed amiodarone	111 (68.1)	25 (54.4)	86 (73.5)	0.02*
Number of failed antiarrhythmics	1.6 ± 1.2	1.5 ± 1.3	1.7 ± 1.2	0.50

Categorical data are presented as numbers and percentages, and continuous data are given as mean ± standard deviation values. *P* values were determined by Student’s *t*-test for continuous variables, and chi-squared/Fisher’s exact tests for categorical variables. A 2-sided *P* < .05 indicates significance.

*Statistically significant.

*Abbreviations:* ACE, angiotensin-converting enzyme inhibitor; ARB, angiotensin receptor blocker; BMI, body mass index; CABG, coronary artery bypass grafting; CAD, coronary artery disease; CMR, cardiac magnetic resonance; COPD, chronic obstructive pulmonary disease; CVA, cerebrovascular accident; LVEF, left ventricular ejection fraction; NYHA, New York Heart Association; PAD, peripheral arterial disease; PCI, percutaneous coronary intervention; P2Y12, P2Y12 inhibitor; VT, ventricular tachycardia.

**Table 2:** Multivariate Regression Analysis for Ventricular Tachycardia Recurrence

Variable	Hazard Ratio	95% Confidence Limits	p-value
Cardiomyopathy (nonischemic vs. ischemic)	2.14	1.31–3.47	0.002
CMR pre-ablation	0.52	0.28–0.96	0.04
Number of failed antiarrhythmics (per drug)	1.49	1.25–1.77	< 0.0001
Female sex	0.32	0.14–0.74	0.007
Ischemia testing	0.99	0.59–1.64	0.96

This table presents the significant predictors (defined as *P* < .05) of VT recurrence on multivariable Cox regression analysis, with ischemia testing added to the model.

*Abbreviations:* CMR, cardiac magnetic resonance; VT, ventricular tachycardia.

**Table 3:** Multivariate Regression Analysis for Ventricular Tachycardia Recurrence in Ischemic Cardiomyopathy Cohort Sub-analysis

Variable	Hazard Ratio	95% Confidence Limits	P-value
Number of failed antiarrhythmics (per drug)	1.64	1.32–2.02	< 0.0001
Female sex	0.20	1.17–21.06	0.02
